# Association between different drinks consumption and risk of inflammatory bowel disease: a dose–response meta–analysis

**DOI:** 10.3389/fnut.2026.1817418

**Published:** 2026-05-20

**Authors:** Yu Jie Peng, Yu Zeng, Ding Niu Li, Ke Xi Wei, Min Li

**Affiliations:** 1 Department of Anorectal Surgery (Integrated Traditional Chinese and Western Medicine), Affiliated Hospital of North Sichuan Medical College, Nanchong, Sichuan, China; 2 College of Clinical Medicine of Integrated Traditional Chinese and Western Medicine, North Sichuan Medical College, Nanchong, Sichuan, China

**Keywords:** alcohol, beverages, coffee, inflammatory bowel disease, meta–analysis, tea

## Abstract

**Objective:**

To systematically review and synthesize the evidence between consumption of different drinks in daily life and the risk of Inflammatory bowel disease (IBD).

**Methods:**

We systematically searched PubMed, Web of Science, SinoMed, Cochrane Library, Embase, Wiley, CNKI, and Wanfang Database for case–control, cohort, and cross-sectional studies investigating the relationship between beverage intake and IBD. A random-effects model was used for the meta-analysis.

**Results:**

A total of 27 articles from 16 regions were included. The meta-analysis showed that soft beverage intake was associated with an increased risk of IBD (OR = 1.144, 95% CI: 1.052–1.243, *p* = 0.002). In contrast, alcohol intake (OR = 0.793, 95% CI: 0.629–0.999, *p* = 0.049), coffee intake (OR = 0.807, 95% CI: 0.667–0.976, *p* = 0.027), and tea intake (OR = 0.711, 95% CI: 0.522–0.970, *p* = 0.031) were associated with a reduced risk of IBD. Dose–response analysis revealed a linear inverse correlation between alcohol (linear trend *p* = 0.011) and coffee (linear trend *p* < 0.001) consumption and IBD risk. For tea consumption a significant nonlinear relationship and a downward trend was found between tea consumption frequency (times/week) and IBD risk (nonlinear trend *p* < 0.001). The risk of IBD showed a slow upward trend within the range of 1.200 to 6.000 times per week.

**Conclusion:**

Soft beverage consumption is associated with the risk of IBD. Current evidence suggests that higher intake of coffee, alcohol, and tea is associated with a reduced risk of IBD; however, further prospective studies are needed to confirm these findings, given the limited number of studies for certain beverages, highly heterogeneity, and the predominance of case–control designs in the current literature.

**Systematic review registation:**

https://inplasy.com/, identifier INPLASY (registration number INPLASY2025120094).

## 1 Introduction

Inflammatory bowel disease (IBD), including ulcerative colitis (UC) and Crohn’s disease (CD), is a chronic inflammatory disorder of the gastrointestinal tract, clinically characterized by abdominal pain, diarrhea, weight loss, and fatigue (1, 2). In recent years, the global prevalence of IBD has continued to rise. Between 1990 and 2017, the global prevalence increase from 79.500 to 84.300 per 100,000 persons, with the total number of patients rising from approximately 3.700 million to 6.800 million. High-income countries such as the United States and the United Kingdom exhibit the highest prevalence rates (464.500 and 449.600 per 100,000 persons, respectively), while regions with lower socioeconomic status generally show lower prevalence (3). Data from New Zealand in 2024 indicate that the prevalence of IBD reached 671 per 100,000 persons, representing an approximate 2.200–fold increase compared to the rate of 308 per 100,000 persons in 2005 (4). The number of IBD patients is projected to reach 1.500 million by 2025 (5). These trends reflect the spread of IBD from traditional high-incidence regions to broader areas, posing a sustained and growing burden on healthcare systems.

The occurrence of IBD symptoms is closely associated with intestinal microbiota dysbiosis. Evidence indicates that the gut microbiota plays a crucial role in both the pathogenesis and progression of IBD (6, 7). In addition, disruption of intestinal barrier function is considered a key factor in the exacerbation of inflammation. Dysregulated immune system responses are regarded as central mechanisms in IBD pathogenesis (8). Both adaptive and innate immune responses are abnormal in IBD patients, potentially leading to persistent intestinal inflammation (1). Furthermore, cytokines, particularly pro-inflammatory cytokines such as interleukin-1 (IL-1), interleukin-6 (IL-6), and tumor necrosis factor (TNF), play an important role in IBD pathogenesis (9). Overexpression of these cytokines may impair intestinal epithelial barrier function, thereby contributing to the exacerbation of disease progression (10).

Diet plays a significant role in IBD management. The inflammatory potential of dietary intake may influence disease progression, and dietary patterns characterized by high inflammatory potential may exacerbate disease activity and associated symptoms (11). For instance, a diet high in red meat has been linked to increased colitis sensitivity and altered gut microbiota, highlighting the importance of dietary optimization in managing IBD risk. Additionally, lifestyle factors such as smoking have been identified as modifiable risk factors for IBD (12). Smoking has a complex relationship with IBD, where it is known to exacerbate CD while having a protective effect against UC. This dichotomy emphasizes the need for personalized lifestyle interventions in IBD management (13).

Given that diet and drinks intake are modifiable lifestyle factors and play a significant role in guiding lifestyle management for patients with IBD, we conducted this meta-analysis to elucidate the association between drinks consumption and the incidence of IBD risk, based on existing observational evidence. Building upon traditional risk factor meta-analysis, this study systematically collected data from multiple prospective cohort studies to perform dose–response analyses. The currently known dietary risk factors for IBD mainly focus on macronutrients in solid foods, while liquid intake is often overlooked or only considered as a secondary variable in the assessment of daily dietary quality. However, beverages are characterized by high frequency of intake and a low satiety masking effect. We identified specific drinks, such as soft beverages, alcohol, coffee, and tea, as potential factors influencing IBD onset. Quantitative models were employed to characterize the dose–response relationship between varying levels of beverages intake (frequency and quantity) and IBD risk. Emphasis was placed on the evaluation of both linear and nonlinear dose–response models to identify critical intake thresholds associated with substantial risk changes and to estimate the magnitude of risk change per unit increase within typical population consumption ranges.

## 2 Materials and methods

### 2.1 Search strategy

A comprehensive literature search was conducted in PubMed, Web of Science, SinoMed, Cochrane Library, Embase, Wiley, CNKI, and Wanfang Academic Journal Full-text Database. The search covered the period from each database’s inception to 2025. The earliest included study was published in 1989; therefore, the actual time span of the analysis is 1989–2025 (Supplementary material 1 for search methods). Additionally, we manually searched existing meta-analyses and review articles to ensure that all relevant studies were included comprehensively.

After completing the search, titles and abstracts were screened to exclude irrelevant studies. The remaining full-text articles underwent rigorous eligibility assessment. Study screening followed the Preferred Reporting Items for Systematic Reviews and Meta-Analyses (PRISMA) guidelines (14). The meta-analysis protocol was registered on INPLASY (https://inplasy.com/: registration number INPLASY2025120094).

### 2.2 Inclusion and exclusion criteria

Inclusion criteria: According to Population, Exposure, Comparator, Outcome, Time frame, Study design (PECOTS) approach.

Participants (P): Adults (≥ 18 years), including both IBDpatients and without non-IBD.

Exposure (E): Consumed at least one of the drinks (soft beverages, alcohol, coffee, tea).

Comparator (C): Lowest vs. highest intake, or non-consumers vs. Consumers.

Outcomes (C): Incident IBD (CD or UC) confirmed by standard criteria.

Time Frame (T): Prospective or retrospective design with clear temporal definition.

Study design (S): cohort, case–control and the cross-sectional study.

Published observational studies reporting consumption data for at least one of the specified beverages (soft beverages, alcohol, coffee, tea) with continuous exposure metrics. Non–consumers were defined as participants reporting zero intake of the specific beverage; consumers were defined as any positive intake. And providing relative risk (RR), odds ratio (OR), or hazard ratio (HR) estimates with corresponding 95% confidence intervals (CI).

Exclusion criteria: (1) Non–research articles, such as reviews, editorials, and conference abstracts; (2) Articles without full-text availability or lacking necessary data; (3) Articles not published in English or Chinese.

### 2.3 Data extraction

Duplicate records were removed by using ZOTERO software. Literature screening and data extraction were performed independently by two reviewers (PYJ and WKX). Discrepancies were resolved through discussion or by a third reviewer (LM). Databases commonly used in contemporary meta–analyses were selected based on relevance and comprehensiveness, including both English and Chinese publications relevant to the topic.

Two reviewers independently read the full texts of included studies and extracted the following data: first author, publication year, study location, study design, number of cases and controls (or cohort size), exposure assessment method, beverage type, consumption frequency, dose metrics, and the reported effect estimates (OR/RR/HR) with 95% CI. The Newcastle-Ottawa Scale (NOS) was used to assess the quality of cohort and case–control studies (15). NOS comprises 9 items, with a score of ≥ 7.000 indicating high quality, 6–4 regard as moderate quality, and ≤ 3.000 low quality. The Agency for Healthcare Research and Quality (AHRQ) tool was used for the single cross-sectional study (16); it consists of 11 items, with scores ≥ 8.000 indicating high quality. The score details of all studies can be found in Supplementary Tables 1, 2.

### 2.4 Statistical analysis

Given the low incidence of IBD, the OR was considered approximate to the RR in this meta-analysis. Since HR and OR are distinct metrics that cannot be interconverted, HR data were not extracted due to insufficient reporting. The OR was used as the effect size for assessing the relationship between drinks consumption and IBD risk. Heterogeneity across studies was evaluated using the I^2^ statistic. If I^2^ ≤ 50% and *p* > 0.050, indicating low heterogeneity, a fixed–effect model was applied. If I^2^ > 50% or *p* ≤ 0.050, indicating substantial heterogeneity, a random-effects model was used, followed by subgroup analysis and meta-regression to explore sources of heterogeneity. Publication bias was assessed using funnel plots and Egger’s test. Sensitivity analysis was performed using the leave-one-out method.

For dose–response meta-analysis, the midpoint of each exposure category was assigned as the representative dose (e.g., 1.500 times/week for the 1.000–2.000 times/week category). For the lowest open–ended category (e.g., ≤ 1.000 time/week), 0.500 times/week was assigned. For the highest open-ended category, the dose was set at 1.200 times the lower bound (e.g., 2.400 times/week for ≥ 2.000 times/week). Dose–response relationships were modeled using the generalized least-squares method for trend estimation (GLST) and restricted cubic splines with three or four knots (positioned at 5, 35, 65, 95% for four knots; 10, 50, 90% for three knots). The significance of linear and nonlinear trends was tested using the Wald test. Statistical analyses were performed using Stata SE12.0 and 15.0 software (Tables 1, 2).

**Table 1:** Characteristics of case–control study and cohort–study.

Study name	Age	Study type	Drink type	Total participants	Patients	Country	Disease type	Gender
Almofarreh 2022 (17)	40.0 ± 12.5	Case–Control	Soft Beverage, Coffee, Tea	571	171	Arab	UC	Both
Chu 2024 (18)	≥ 18	Case–Control	Alcohol	1,230	563	China	IBD	Both
Boyko 1989 (19)	18–65	Case–Control	Coffee, Alcohol	418	209	Washington	UC	NA
Cui 2018 (20)	17–67	Case–Control	Alcohol	94	47	China	CD	Both
DugYeoHan 2010 (21)	5–86	Case–Control	Alcohol	851	315	NewZealand	CD	Both
Zhang 2012 (22)	13–55	Case–Control	Tea	128	64	China	CD	Both
Hammer 2019 (23)	11–67	Cohort- Study	Alcohol	5,698	37	Faroese	IBD	NA
Jowett 2004 (24)	18–70	Prospective- Cohort Study	Soft Beverage	191	191	England	UC	Both
Jakobsen 2013 (25)	2.9–14.9	Case–Control	Soft Beverage	595	118	Denmark	IBD	Both
Hansen 2011 (26)	10–95	Case–Control	Coffee	534	267	Copenhagen	IBD	Both
Nakamura 1994 (27)	NA	Case–Control	Alcohol	768	384	Japan	UC	Both
Niewiadoski 2016 (28)	11–76	Case–Control	Coffee	236	132	Barwon	IBD	Both
Li 2007 (29)	9–82	Case–Control	Alcohol, Tea	354	177	China	UC	Both
Kondo 2022 (30)	8.7–74.8	Case–Control	Alcohol	299	132	Japan	UC	Both
Russel 1998 (31)	CD:21–38 UC:27–51	Case–Control	Soft Beverage, Coffee, Tea	1,304	688	Limburg	IBD	Both
Siew 2014 (32)	Median:38	Prospective Case–Control	Soft Beverage, Coffee, Tea	1,382	442	Asia and Australia	IBD	Both
Niu 2016 (33)	UC:46.45 ± 15.74 CD:39.03 ± 16.26	Nested Case–Control	Tea	3,900	780	China	IBD	Both
Salih 2017 (34)	NA	Prospective Case–Control	Alcohol	525	98	Vasterbotten	IBD	NA
Bergmann 2017 (35)	UC:23.2–77.1 CD:23.0–75.8	Prospective Cohort- Study	Alcohol	1,410	282	European	IBD	Both
Tanaka 2024 (36)	Median:43	Case–Control Study	Tea, Coffee	1,049	384	Japan	UC	Both
Sun 2025 (37)	35.16 ± 7.62	Case–Control	Soft Beverage, Tea	318	159	China	IBD	Both
Liu 2022 (38)	NA	Prospective Cohort- Study	Alcohol	502,505	7,095	UK	IBD	Both
Hart 2008 (39)	20–80	Prospective Cohort- Study	Alcohol	260,686	139	European	UC	NA
Wang 2013 (40)	16–70	Case–Control	Tea	2,616	1,308	China	UC	Both
Sakamoto 2005 (41)	15–34	Case–Control	Alcohol	445	108	Japan	IBD	Both
Persson 1992 (42)	15–79	Case–Control	Coffee	602	152	Stockholm	IBD	Both

**Table 2:** Characteristics of cross–sectional study.

Study name	Age	Study type	Beverage type	Total participants	Patients	Country	Disease type	Gender
Han 2020 (64)	18–85	Cross-Sectional Study	Soft Beverage, Coffee/Tea	33,672	454	US	IBD	Both

## 3 Results

### 3.1 Study characteristics

**Figure 1:**
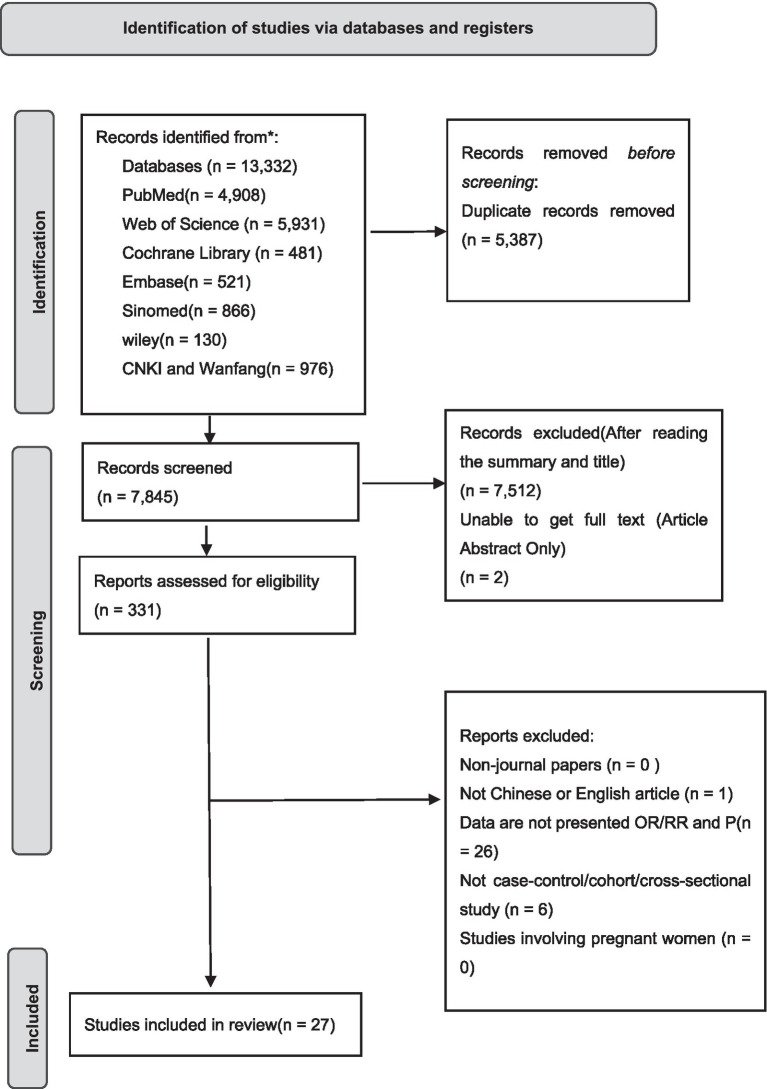
Document screening process and results.

A total of 13,332 records were retrieved. After removal of duplicates and application of the inclusion and exclusion criteria, a total of 331 articles were retained for title and abstract screening. Following full-text review, 27 articles were included totally (17–42), encompassing 822,381 participants, among whom 14,896 were diagnosed with IBD. In the included studies, 22 were case–control, 4 were cohort, and 1 was a cross-sectional study. The literature screening process is presented in Figure 1.

### 3.2 Quality assessment

**Figure 2:**
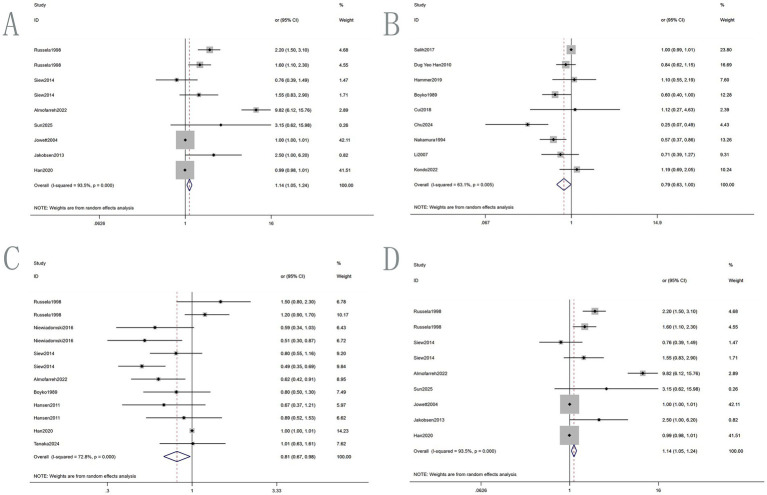
Forest plot: relationship between IBD and different drinks. **(A)** Beverage; **(B)** alcohol; **(C)** coffee; **(D)** tea.

The included cohort and case–control studies scored 7.73, while the cross–sectional study scored 8.00 (Supplementary Tables 1, 2 for details; Figure 2).

### 3.3 Soft beverages

**Figure 3:**
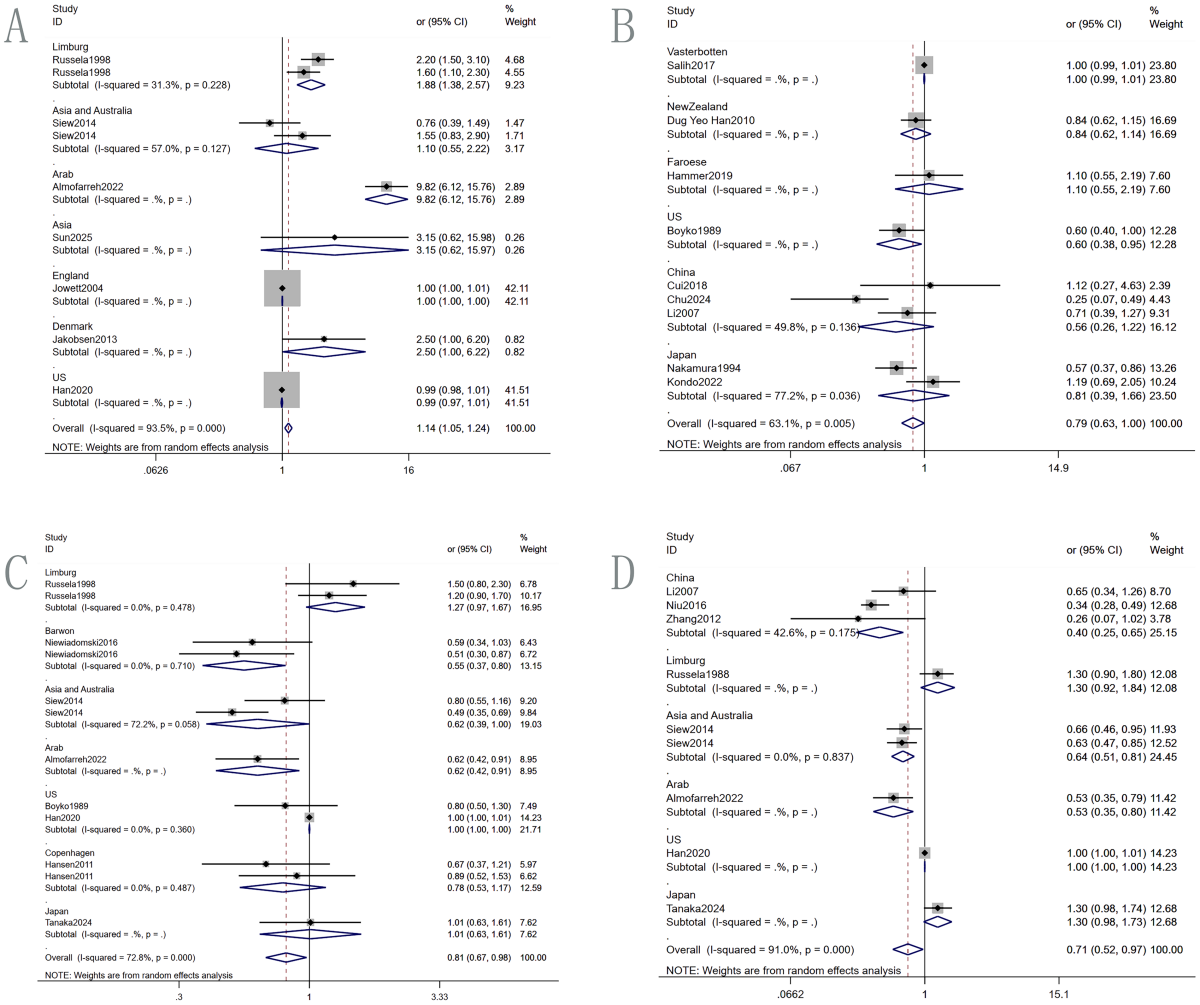
Forest plot of regional subgroup: relationship between different drinks. **(A)** Beverage; **(B)** alcohol; **(C)** coffee; **(D)** tea.

**Figure 4:**
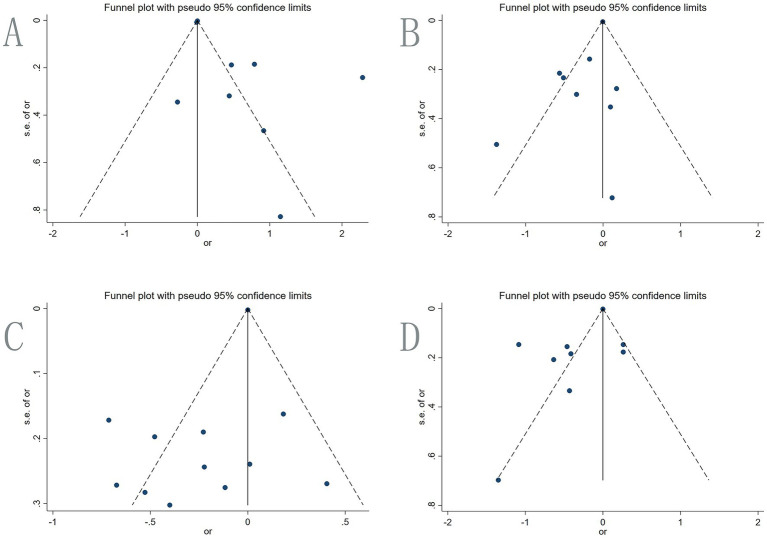
Funnel plot. **(A)** Beverage; **(B)** alcohol; **(C)** coffee; **(D)** tea.

**Figure 5:**
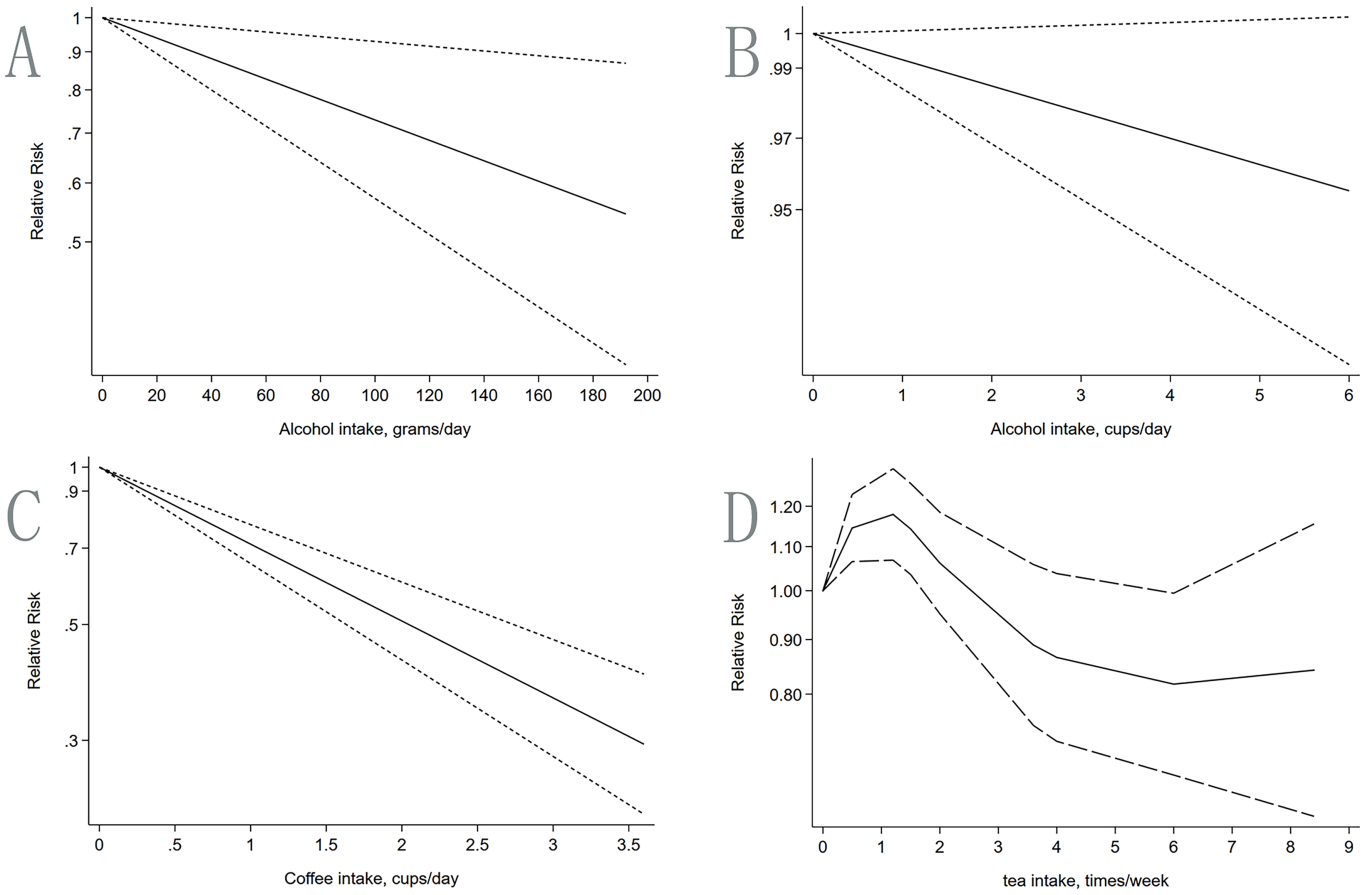
Dose–response: relationship between different drinks intake and the risk of IBD. **(A,B)** Alcohol; **(C)** Coffee; **(D)** Tea.

Seven multivariate studies involving 2,223 IBD patients explored the association between soft beverage intake and IBD risk (Figure 3A; Table 3). The random-effects meta-analysis showed a 14.4% increased risk of IBD among consumers compared to non-consumers (OR = 1.144, 95% CI: 1.052–1.243, *p* = 0.002). Substantial heterogeneity was observed (I^2^ = 93.5%). Subgroup analysis by region (Figure 4A) revealed a significant positive association in Arab countries (OR = 9.820, 95% CI: 6.119–15.758, *p* < 0.001), but no significant association in the United States (OR = 0.990, 95% CI: 0.974–1.006, *p* = 0.223). This discrepancy may be attributed to differences in genetic background, dietary patterns, or masking by other stronger risk factors. Funnel plot inspection suggested asymmetry (Figure 5A), but Egger’s test did not indicate significant publication bias (*p* = 0.065; Supplementary material S1). Sensitivity analysis using the leave-one-out method showed that excluding the study by Han et al. yielded the highest pooled OR (1.995, 95% CI, 1.711–2.327; Supplementary material S5).

**Table 3:** Summary of influencing factor results.

Intervention	Number of studies included (articles)	Sample size (n)	Heterogeneity	Model selection	Merge effect quantity OR(95%CI)	*p* value	Egger test
Soft Beverage	7	38,033	93.50%	Random	1.144 (1.052–1.243)	*p* = 0.002	0.065
Alcohol	9	10,237	63.10%	Random	0.793 (0.629–0.999)	*p* = 0.049	0.059
Coffee	8	39,166	72.80%	Random	0.738 (0.667–0.976)	*p* = 0.027	0.051
Tea	8	42,360	91.00%	Random	0.403 (0.250–0.650)	*p* = 0.000	0.098

### 3.4 Alcohol

Nine multivariate studies involving 1,962 IBD patients examined the association between alcohol intake and IBD risk. The pooled random-effects analysis indicated a reduced risk among drinkers compared to non-drinkers (OR = 0.793, 95% CI: 0.629–0.999, *p* = 0.049; Figure 3B; Table 3). This result differed from those reported in previous studies. Heterogeneity was moderate (I^2^ = 63.1%). Subgroup analysis by region (Figure 4B) showed non-significant associations in the Faroe Islands (OR = 1.100, 95% CI: 0.550–2.190, *p* = 0.787) and China (OR = 0.562, 95% CI: 0.260–1.216, *p* = 0.143). Funnel plot inspection suggested asymmetry (Figure 5B), but Egger’s test did not indicate significant publication bias (*p* = 0.059; Supplementary material S2). Sensitivity analysis (Supplementary material S6) showed pooled OR ranging from 0.739 to 0.845 upon sequential exclusion of individual studies. Excluding Salih et al. resulted in an OR = 0.738 (95% CI: 0.570–0.956), without fundamentally altering the overall conclusion.

### 3.5 Coffee

Eight studies involving 2,747 IBD patients investigated the association between coffee consumption and IBD risk. The pooled random-effects analysis showed a 19.3% lower risk of IBD among coffee drinkers compared to non-drinkers (OR = 0.807, 95% CI: 0.667–0.976, *p* = 0.027), suggesting a protective association (Figure 3C; Table 3). Heterogeneity was substantial, with an I^2^ value of 72.8%. Subgroup analysis by region using a random–effects model found significant inverse associations in Barwon (Figure 4C), Asia and Australia, and Arab regions (e.g., Barwon OR = 0.547, 95% CI: 0.373–0.803). Positive associations were observed in the US and Japan, which may be due to regional differences in coffee consumption frequency, preparation methods, or bean composition. Egger’s test did not detect significant reporting bias (*p* = 0.051). The funnel plot showed some asymmetry (Figure 5C). Sensitivity analysis (Supplementary material S7) showed the lowest pooled OR = 0.770 (95% CI: 0.632–0.940) after excluding the two studies by Russel et al.

### 3.6 Tea

Eight studies involving 3,160 IBD patients examined the association between tea consumption and IBD risk. Tea drinkers had a 28.9% lower risk of IBD compared to non-drinkers (OR = 0.711, 95% CI: 0.522–0.970, *p* = 0.031), indicating a significant inverse association (Figure 3D; Table 3). Heterogeneity was high (I^2^ = 91.0%). Subgroup analysis by region (Figure 4D) using a random-effects model showed significant risk reductions in China (OR = 0.403, 95% CI: 0.250–0.650), Asia/Australia (OR = 0.643, 95% CI: 0.510–0.811), and the Arab region (OR = 0.530, 95% CI: 0.353–0.796). Positive associations were observed in other regions. Egger’s test did not identify significant reporting bias (*p* = 0.098; Supplementary material S4). The funnel plot showed some asymmetry (Figure 5D). Sensitivity analysis (Supplementary material S8) showed the highest pooled OR = 0.822 (95% CI: 0.630–1.074) after excluding Niu et al.

## 4 Dose–response analysis

From the above analysis, differences exist in the association between various drinks and IBD risk. However, current studies primarily focus on drinks consumption, and the dose–response relationship between intake levels and disease incidence has not been systematically explored. Therefore, we summarized studies reporting dose categories with continuous multiple and conducted dose–response meta-analyses. Due to insufficient dose–response data for soft beverages, this portion of the dataset was excluded because the extremely low case count precluded stable dose–response model fitting.

Alcohol: A linear inverse relationship was found between alcohol consumption (g/day) and IBD risk (linear trend *p* = 0.011). Eight studies were included. For every 40 g/day increase in alcohol intake, the risk of IBD decreased by 12% (Figure 5A). Analysis using cups/day showed no significant association (linear *p* = 0.076; nonlinear *p* = 0.686), but the trend remained consistent with the above unit analysis, i.e., higher alcohol intake was associated with lower IBD risk (Supplementary Tables 3, 4 for details). For a 1.500 cups/day increase in alcohol consumption, the risk of IBD decreased by 1% (Figure 5B). This finding notably deviated from conventional understanding and required further prospective investigation for validation.

Coffee: A significant linear inverse relationship was observed between coffee intake (cups/day) and IBD risk (linear trend *p* < 0.001). Three studies were included. The higher the coffee intake, the lower the IBD risk. Specifically, for each additional cup per day, the risk of IBD decreased by 29% (Figure 5C; Supplementary Table 5). This suggests that moderate coffee consumption in daily life may help to reduce a risk of IBD.

Tea: A significant nonlinear relationship was found between tea consumption frequency (times/week) and IBD risk (nonlinear trend *p* < 0.001). Three studies were included. When tea consumption was 0.000–1.200 times/week, IBD risk was positively correlated with tea intake, and risk increased with increasing consumption. Specifically, within 0.000–0.500 times/week, risk increased by 15% per 0.500 times/week increment. Within 0.500–1.200 times/week, risk increased 3% per 0.700 times/week increment. When tea consumption was > 1.200 times/week but < 6.000 times/week, IBD risk was negatively correlated with tea intake. Specifically, within 1.500–3.5999 times/week, risk decreased by 17% per 2.099 times/week increment. Within 4.000–6.000 times/week, risk decreased 5% per 2.000 times/week increment. However, tea consumption exceeding 6.000 times/week gradually was again positively associated with IBD risk (Supplementary Table 6 for details). The overall trend was an initial increase, followed by a decrease, and then a slow increase (Figure 5D). This suggests that tea consumption may reduce IBD risk within a certain range.

### 4.1 Discussion of heterogeneity

The observed fluctuation in the association of soft beverage consumption suggests that heterogeneity may be related to the cross-sectional design of Han et al., which may be susceptible to recall bias and issues of causal timing.

The alcohol group incorporates the study by Salih et al., which enrolled middle-aged and older adults (aged 40, 50, and 60 years at enrollment) diagnosed with late-onset IBD (median age at diagnosis 52–53 years), and this may be a source of heterogeneity.

When excluding the two studies by Russel et al., 1998 in the coffee group, sensitivity analysis showed the lowest pooled OR. This may be due to their broad exposure definition (“daily consumption” vs. “non-daily consumption”) and specific distinction between pre-symptomatic and post-diagnosis consumption habits, with the latter potentially influenced by the disease.

The tea subgroup excluding Niu et al. was analyzed separately, as Niu et al. was conducted in a plateau region of Southwest China via an internet-based nested case–control study, whereas the other Chinese studies (e.g., Li et al. from Wuhan and Zhang et al. from Shaanxi) were conducted in different, more urbanized geographical settings using offline data collection. Additionally, Niu et al. clearly distinguished between UC and CD and reported tea consumption only for UC patients.

### 4.2 Mechanistic considerations

Certain components in coffee may influence the pathogenesis of IBD by modulating the composition of gut microbiota. Studies have shown that long-term coffee consumption is associated with alterations in human fecal microbiota composition, which may be related to polyphenols and alkaloids present in coffee (43). These microbial changes could potentially modulate intestinal inflammatory states, thereby exerting a certain degree of influence on IBD development. Second, certain components in coffee may influence the pathogenesis of IBD by inducing nuclear translocation of NF-κB in intestinal tissues. NF-κB serves as a key factor in the intestinal immune system, and IBD is associated with chronic activation of NF-κB. Studies have indicated that coffee and its roasted products can induce nuclear translocation of NF-κB in intestinal tissues, an effect potentially linked to roasted coffee compounds (44). This mechanism may contribute to IBD development by exacerbating intestinal inflammatory responses. Furthermore, coffee consumption may influence the pathogenesis of IBD by affecting intestinal inflammatory markers. In one study, fecal calprotectin levels were significantly lower in IBD patients who consumed coffee compared to non-consumers, particularly in patients with ulcerative colitis, suggesting that coffee may affect IBD onset by modulating intestinal inflammatory responses (45). Finally, although some studies have failed to establish a direct causal relationship between coffee consumption and IBD, certain components in coffee, such as caffeine, may indirectly influence the pathogenesis of IBD by modulating inflammatory responses (46).

The relationship between alcohol intake and IBD is complex, with dual effects. Studies indicate that alcohol intake may influence the onset of IBD through multiple mechanisms. First, alcohol consumption can lead to alterations in gut microbiota and disruption of intestinal barrier function, thereby increasing intestinal permeability. These changes facilitate the entry of bacterial components and metabolites into the systemic circulation, triggering immune system activation and inflammatory responses (47, 48). However, specific components of alcohol or low-dose intake may exhibit limited anti–inflammatory effects, offer potential benefits for the prevention of IBD. Underlying mechanisms include alcohol-induced changes in gut flora, disruption of the intestinal mucosal barrier, and increased intestinal permeability, thereby promoting immune activation and inflammation (47). At the genetic level, genome-wide association study (GWAS) data suggest a significant correlation between alcohol intake and IBD, particularly CD (49), indicating genetic factors may play a role. The effect of alcohol is not uniform and likely depends on dose and beverage type. For example, in UC patients, low-to-moderate alcohol consumption was associated with a smaller extent of lesions (50). Additionally, antioxidant components such as resveratrol in red wine have anti-inflammatory effects and inhibit neutrophil migration, theoretically helping alleviate inflammation (51). However, despite red wine’s potential anti–inflammatory properties, long-term moderate daily intake may still increase the risk in IBD patients (52).

The mechanism of tea consumption in IBD is complex and multifaceted. Active components in tea, such as polyphenols, tea polysaccharides, and tea pigments, are believed to possess anti-inflammatory and antioxidant properties, which may offer potential benefits for the prevention and treatment of IBD (53). Notably, tea polysaccharides have also been demonstrated to exert positive effects in regulating intestinal immunity and microbiota. For instance, polysaccharides from Tieguanyin Oolong tea exhibited significant protective effects in a mouse colitis model by modulating T-cell-mediated immune responses, improving intestinal metabolism, and increasing short-chain fatty acid levels, thereby demonstrating potential for the prevention and treatment of inflammatory bowel disease (IBD) (54). Additionally, endogenous polysaccharides in Fuzhuan tea enhance intestinal microbiota balance by promoting the enrichment of beneficial bacteria such as Bacteroides and Fecalibacterium, which prevents inflammation and repairs intestinal barrier function (55).

High intake of sugar and soft beverages may be associated with increased IBD risk (56). Multiple studies consistently link soft beverage consumption to elevated IBD risk. A systematic review and meta-analysis by Maniyar et al., found significant associations between soft beverage intake and the risk of developing CD and UC (57). A study by Fu et al. also suggested an association with increased IBD risk, though the trend was not significant (58). High consumption is also linked to increased disease severity and inflammatory markers in IBD patients, and different beverage types may have varying effects (59). The impact of beverage consumption on the gut microbiome may also be an important factor in IBD pathogenesis. Shon et al., demonstrated that combined consumption of soft beverages and a high-fat diet significantly alters gut microbiome composition and increases the relative abundance of IBD-associated pathogenic bacteria, exacerbating intestinal inflammation (60). This suggests beverage consumption may influence IBD pathogenesis via gut microbiota modulation. Although the present study found an association between beverages and increased IBD risk, a study by Khalili et al., found no association with intake of one or more servings per day (61).

### 4.3 Clinical implications

This study subcategorizes daily beverages into soft beverages, alcohol, coffee, tea, and others, aiding clinicians in more precisely assessing the role of different beverages in patient rehabilitation. This distinction has practical significance for dietary guidance in IBD patients, enabling more personalized and scientific dietary advice tailored to different conditions and disease stages, thereby better supporting clinical treatment and rehabilitation management.

### 4.4 Comparison with previous studies

Piovani et al.’s systematic review evaluated the association of 71 environmental factors with IBD, including beverages such as soft drinks, tea, alcohol, and coffee, and UC incidence. The study graded evidence strength but did not separately discuss beverages or conduct dose–response analyses, focusing only on UC patient data (62). Gao et al., focused on UC, finding soft drinks increased UC risk and tea had a protective effect, and conducted subgroup analyses (racial differences), but did not include CD or dose–response assessment (63). Yang et al., conducted a meta-analysis specifically on soft drinks, suggesting associations with both UC and CD risk, but the number of studies was limited, heterogeneity was high, and again dose–response was not explored (56). This study analyzed “beverages” high in sugar, consistent with Maniyar et al.’s conclusion of a positive correlation (57). However, the current study more systematically evaluated four common beverages (soft beverages, alcohol, coffee, tea) and conducted dose–response meta-analyses. The core contribution is the preliminary revelation of a linear inverse relationship between alcohol/coffee intake and IBD risk, and a nonlinear relationship for tea consumption, providing more clinically relevant “dose–response” information beyond simple “correlation.”

### 4.5 Limitations and future directions

Limitations include: (1) All included studies were observational, so confounding cannot be completely excluded; (2) Measurement errors due to inconsistent methods for assessing beverage exposure across studies may affect result reliability; (3) Restriction to English and Chinese articles may introduce selection bias.

In addition, these studies utilized diverse dietary assessment instruments, the number and granularity of consumption categories, and recall timeframes. Such inter-study variability likely contributed to non-differential exposure misclassification. This measurement heterogeneity warrants careful consideration during interpretation of the findings. Further studies are needed, and prospective trials should be designed considering different regional dietary patterns to extend current understanding.

## 5 Summary

In this meta-analysis, we synthesized 27 observational studies from 16 regions. We systematically evaluated the association between intake of four common drinks (soft beverages, alcohol, coffee, and tea) and IBD risk. Results suggest that reasonable consumption of coffee, tea, and moderate alcohol in the daily diet may help reduce IBD risk. Dose–response analyses indicated inverse linear relationships for alcohol and coffee intake with IBD risk, i.e., risk further decreased with increasing intake. Tea intake showed a nonlinear relationship with IBD risk, exhibiting a protective effect at moderate intake (1.200–6.000 times/week), but opposite effects at excessive or very low intake.

Although this study employed subgroup analysis, sensitivity analysis, and publication bias testing, limitations remain, including the observational nature of included studies, inconsistent measurement methods for beverage intake, and potential regional or population selection bias. There might also be differences between the two IBD subtypes, as some articles did not clearly indicate whether the study population had UC or CD. This might have affected the analysis of heterogeneity sources. Furthermore, further experimental studies are needed to clarify the mechanisms of different beverages.

## Data Availability

The original contributions presented in the study are included in the article/Supplementary material, further inquiries can be directed to the corresponding author.

## References

[1] Rubin DC Shaker A Levin MS. Chronic intestinal inflammation: inflammatory bowel disease and colitis–associated colon cancer. Front Immunol. (2012) 3:107. doi: 10.3389/fimmu.2012.00107, 22586430 PMC3347037

[2] Bruner LP White AM Proksell S. Inflammatory bowel disease. Prim Care. (2023) 50:411–27. doi: 10.1016/j.pop.2023.03.009, 37516511

[3] Alatab S Sepanlou SG Ikuta K. The global, regional, and national burden of inflammatory bowel disease in 195 countries and territories, 1990–2017: a systematic analysis for the global burden of disease study 2017. Lancet Gastroenterol Hepatol. (2020) 5:17–30. doi: 10.1016/S2468-1253(19)30333-4 31648971 PMC7026709

[4] Forbes AJ Day AS Frampton CMA Gearry RB. Compounding prevalence of inflammatory bowel disease in a 2024 population-based study from Canterbury, New Zealand. JGH Open. (2025) 9:e70192. doi: 10.1002/jgh3.70192, 40452912 PMC12125484

[5] Kaplan GG Windsor JW. The four epidemiological stages in the global evolution of inflammatory bowel disease. Nat Rev Gastroenterol Hepatol. (2021) 18:56–66. doi: 10.1038/s41575-020-00360-x, 33033392 PMC7542092

[6] Hassouneh SA–D Loftus M Yooseph S. Linking inflammatory bowel disease symptoms to changes in the gut microbiome structure and function. Front Microbiol. (2021) 12:673632. doi: 10.3389/fmicb.2021.673632, 34349736 PMC8326577

[7] Jauregui–Amezaga A Smet A. The microbiome in inflammatory bowel disease. J Clin Med. (2024) 13:4622. doi: 10.3390/jcm13164622, 39200765 PMC11354561

[8] Uwada J Nakazawa H Muramatsu I Masuoka T Yazawa T. Role of muscarinic acetylcholine receptors in intestinal epithelial homeostasis: insights for the treatment of inflammatory bowel disease. Int J Mol Sci. (2023) 24:6508. doi: 10.3390/ijms24076508, 37047478 PMC10095461

[9] Polińska B Matowicka–Karna J Kemona H. The cytokines in inflammatory bowel disease. Postepy Hig Med Dosw. (2009) 63:389–94. Available online at: https://pubmed.ncbi.nlm.nih.gov/19724079/19724079

[10] Fakhoury M Negrulj R Mooranian A H A–S. Inflammatory bowel disease: clinical aspects and treatments. J Inflamm Res. (2014) 7:113–20. doi: 10.2147/JIR.S65979, 25075198 PMC4106026

[11] Anneberg OM Petersen ISB Jess T de Freitas MB Jalili M. The dietary inflammatory potential and its role in the risk and progression of inflammatory bowel disease: a systematic review. Clin Nutr. (2025) 47:146–56. doi: 10.1016/j.clnu.2025.02.019, 40022954

[12] Huang S Yang K Sun D Pan L Ma L Li M . Red meat diet exacerbates colitis by promoting the accumulation of myeloid cells and disrupting gut microbiota. Mol Nutr Food Res. (2025) 69:e70203. doi: 10.1002/mnfr.70203, 40830041

[13] Hu W Li SY Luo JJ. Declining bowel surgery rates and predictors for surgery of Crohn’s disease: a prospective inception cohort in eastern China. J Dig Dis. (2025) 26:348–58. doi: 10.1111/1751-2980.70002 40831043

[14] Page MJ McKenzie JE Bossuyt PM Boutron I Hoffmann TC Mulrow CD . The PRISMA 2020 statement: an updated guideline for reporting systematic reviews. Syst Rev. (2021) 10:1–11. doi: 10.1186/s13643-021-01626-4 33781348 PMC8008539

[15] Gualdi-Russo E Zaccagni L. The newcastle–ottawa scale for assessing the quality of studies in systematic reviews. Publications. (2026) 14:4. doi: 10.3390/publications14010004

[16] Sprinz C Altmayer S Zanon M Watte G Irion K Marchiori E . Effects of blood glucose level on 18F-FDG uptake for PET/CT in normal organs: A systematic review. PLoS ONE. (2018) 13:e0193140. doi: 10.1371/journal.pone.0193140 29486008 PMC5828444

[17] Almofarreh A Sheerah HA Arafa A Ahamed SS Alzeer O al-Hunaishi W . Beverage consumption and ulcerative colitis: a case–control study from Saudi Arabia. Int J Environ Res Public Health. (2022) 19:2287. doi: 10.3390/ijerph19042287, 35206479 PMC8872579

[18] Chu X Chen X Zhang H Wang Y Guo H Chen Y . Association of diet and outdoor time with inflammatory bowel disease: a multicenter case–control study using propensity matching analysis in China. Front Public Health. (2024) 12:1368401. doi: 10.3389/fpubh.2024.1368401, 38952728 PMC11215971

[19] Boyko EJ Perera DR Koepsell TD. Coffee and alcohol use and the risk of ulcerative colitis. Am J Gastroenterol. (1989) 84:530–4. 2719009

[20] Cui DJ Yang LC Yang XL Han R Yuan W Zhang M. Risk factors of Crohn's disease in Guizhou population. J Clin Pathol Res. (2018) 38:1913–6. doi: 10.3978/j.issn.2095-6959.2018.09.016

[21] Han DY Fraser AG Dryland P Ferguson LR. Environmental factors in the development of chronic inflammation: a case–control study on risk factors for crohn’s disease within New Zealand. Mutat Res. (2010) 690:116–22. doi: 10.1016/j.mrfmmm.2009.09.002, 19751746

[22] Zhang Wei ZW Dong Tao DT Liu ZhenZhen LZ Han Shuang HS Liang ShuHui LS Wang BiaoLuo WB . A case–control study on the risk factors of Crohn's disease in Shaanxi population. Progress in Modern Biomedicine (2012) 12:6674–6676. doi: 10.13241/j.cnki.pmb.2012.34.027,

[23] Hammer T Lophaven SN Nielsen KR Petersen MS Munkholm P Weihe P . Dietary risk factors for inflammatory bowel diseases in a high–risk population: results from the faroese IBD study. United European Gastroenterol J. (2019) 7:924–32. doi: 10.1177/2050640619852244, 31428417 PMC6683641

[24] Jowett SL Seal CJ Pearce MS Phillips E Gregory W Barton JR . Influence of dietary factors on the clinical course of ulcerative colitis: a prospective cohort study. Gut. (2004) 53:1479–84. doi: 10.1136/gut.2003.024828, 15361498 PMC1774231

[25] Jakobsen C Paerregaard A Munkholm P Wewer V. Environmental factors and risk of developing paediatric inflammatory bowel disease –– a population based study 2007–2009. J Crohns Colitis. (2013) 7:79–88. doi: 10.1016/j.crohns.2012.05.024, 22748696

[26] Hansen TS Jess T Vind I Elkjaer M Nielsen MF Gamborg M . Environmental factors in inflammatory bowel disease: a case–control study based on a danish inception cohort. J Crohns Colitis. (2011) 5:577–84. doi: 10.1016/j.crohns.2011.05.010, 22115378

[27] Nakamura Y Labarthe DR. A case–control study of ulcerative colitis with relation to smoking habits and alcohol consumption in Japan. Am J Epidemiol. (1994) 140:902–11. 7977277 10.1093/oxfordjournals.aje.a117178

[28] Niewiadomski O Studd C Wilson J Williams J Hair C Knight R . Influence of food and lifestyle on the risk of developing inflammatory bowel disease. Intern Med J. (2016) 46:669–76. doi: 10.1111/imj.13094, 27059169

[29] Jiang L Xia B Li J Ye M Deng C Ding Y . Risk factors for ulcerative colitis in a Chinese population: an age–matched and sex–matched case–control study. J Clin Gastroenterol. (2007) 41:280–4. doi: 10.1097/01.mcg.0000225644.75651.f1, 17426467

[30] Kondo K Ono Y Ohfuji S Watanabe K Yamagami H Watanabe M . Smoking and drinking habits relating to development of ulcerative colitis in japanese: a multicenter case–control study. JGH Open. (2023) 7:61–7. doi: 10.1002/jgh3.12857, 36660047 PMC9840195

[31] Russel MG Engels LG Muris JW. Modern life’ in the epidemiology of inflammatory bowel disease: a case–control study with special emphasis on nutritional factors. Eur J Gastroenterol Hepatol. (1998) 10:243–9. 9585029 10.1097/00042737-199803000-00010

[32] Ng SC Tang W Leong RW. Environmental risk factors in inflammatory bowel disease: a population–based case–control study in asia–pacific. Gut. (2015) 64:1063–71. doi: 10.1136/gutjnl-2014-307410 25217388

[33] Niu J Miao J Tang Y Nan Q Liu Y Yang G . Identification of environmental factors associated with inflammatory bowel disease in a southwestern highland region of China: a nested case–control study. PLoS One. (2016) 11:e0153524. doi: 10.1371/journal.pone.0153524, 27070313 PMC4829194

[34] Salih A Widbom L Hultdin J Karling P. Smoking is associated with risk for developing inflammatory bowel disease including late onset ulcerative colitis: a prospective study. Scand J Gastroenterol. (2018) 53:173–8. doi: 10.1080/00365521.2017.1418904, 29262738

[35] Bergmann MM Hernandez V Bernigau W Boeing H Chan SSM Luben R . No association of alcohol use and the risk of ulcerative colitis or crohn’s disease: data from a european prospective cohort study (EPIC). Eur J Clin Nutr. (2017) 71:566. doi: 10.1038/ejcn.2017.16, 28377581

[36] Tanaka K Okubo H Miyake Y Nagata C Furukawa S Andoh A . Coffee and caffeine intake reduces risk of ulcerative colitis: a case–control study in Japan. J Gastroenterol Hepatol. (2024) 39:512–8. doi: 10.1111/jgh.16439, 38073066

[37] Sun HY Fan XY Zhang ZY. Influencing factors of inflammatory bowel diseaseand its correlation with dietary intake. Clin Medical Res Prac. (2025) 10:51–4. doi: 10.19347/j.cnki.2096-1413.202526013

[38] Liu B–X Yang J Zeng C Dai X-J Chen Y. Risk of inflammatory bowel disease appears to vary across different frequency, amount, and subtype of alcoholic beverages. Front Nutr. (2022) 9:918754. doi: 10.3389/fnut.2022.918754, 35967782 PMC9363781

[39] Hart AR Luben R Olsen A Tjonneland A Linseisen J Nagel G . Diet in the aetiology of ulcerative colitis: a european prospective cohort study. Digestion. (2008) 77:57–64. doi: 10.1159/000121412, 18349539

[40] Wang Y–F Ou–Yang Q Xia B. Multicenter case–control study of the risk factors for ulcerative colitis in China. World J Gastroenterol. (2013) 19:1827–33. doi: 10.3748/wjg.v19.i11.1827 23555172 PMC3607760

[41] Sakamoto N Kono S Wakai K. Dietary risk factors for inflammatory bowel disease: a multicenter case–control study in Japan. Inflamm Bowel Dis. (2005) 11:154–63. doi: 10.1097/00054725-200502000-00009 15677909

[42] Persson PG Ahlbom A Hellers G. Diet and inflammatory bowel disease: a case–control study. Epidemiology. (1992) 3:47–52. 1313310 10.1097/00001648-199201000-00009

[43] González S Salazar N Ruiz–Saavedra S Gómez-Martín M de los Reyes-Gavilán CG Gueimonde M. Long–term coffee consumption is associated with fecal microbial composition in humans. Nutrients. (2020) 12:1287. doi: 10.3390/nu12051287, 32369976 PMC7282261

[44] Sauer T Raithel M Kressel J Muscat S Münch G Pischetsrieder M. Nuclear translocation of NF–κB in intact human gut tissue upon stimulation with coffee and roasting products. Food Funct. (2011) 2:529–40. doi: 10.1039/c1fo10055f, 21904755

[45] Neamți L Gheorghe SR Ventuneac A Drugan T Drugan C Silaghi CN . Impact of coffee consumption on subjective perception and inflammatory markers in patients with inflammatory bowel diseases. Biomedicine. (2024) 12:1733. doi: 10.3390/biomedicines12081733, 39200197 PMC11351584

[46] Georgiou AN Ntritsos G Papadimitriou N Dimou N Evangelou E. Cigarette smoking, coffee consumption, alcohol intake, and risk of crohn’s disease and ulcerative colitis: a mendelian randomization study. Inflamm Bowel Dis. (2021) 27:162–8. doi: 10.1093/ibd/izaa152, 32628751

[47] White BA Ramos GP Kane S. The impact of alcohol in inflammatory bowel diseases. Inflamm Bowel Dis. (2022) 28:466–73. doi: 10.1093/ibd/izab089, 33988227

[48] Stärkel P Leclercq S de Timary P Schnabl B. Intestinal dysbiosis and permeability: the yin and yang in alcohol dependence and alcoholic liver disease. Clin Sci (Lond). (2018) 132:199–212. doi: 10.1042/CS20171055, 29352076

[49] Huang Z Yuan W. Exploring genetic structures and shared sites between alcohol, cheese intake, and inflammatory bowel disease. Front Nutr. (2025) 12:1468457. doi: 10.3389/fnut.2025.1468457, 39917747 PMC11798781

[50] Martinho–Grueber M Kapoglou I Bravo F Sarraj R Benz E Restellini S . Alcohol and cannabis consumption in patients with inflammatory bowel disease: prevalence, pattern of consumption and impact on the disease. Eur J Gastroenterol Hepatol. (2023) 35:21–30. doi: 10.1097/MEG.0000000000002453, 36317770 PMC9719838

[51] Patel M Keshavarzian A Kottapalli V. Human neutrophil functions are inhibited in vitro byclinically relevant ethanol concentrations. Alcohol Clin Exp Res. (1996) 20:275–83. doi: 10.1111/j.1530-0277.1996.tb01640.x 8730218

[52] Swanson GR Tieu V Shaikh M Forsyth C Keshavarzian A. Is moderate red wine consumption safe in inactive inflammatory bowel disease? Digestion. (2011) 84:238–44. doi: 10.1159/000329403, 21876358 PMC3180655

[53] Huang Y Xing K Qiu L Wu Q Wei H. Therapeutic implications of functional tea ingredients for ameliorating inflammatory bowel disease: a focused review. Crit Rev Food Sci Nutr. (2022) 62:5307–21. doi: 10.1080/10408398.2021.1884532, 33635174

[54] Zhang Y Pan Y Lin H Chen X He P Wang Y . Crude tieguanyin oolong tea polysaccharides regulate intestinal immune and gut microflora in dextran sulfate sodium–induced mice colitis. J Sci Food Agric. (2024) 104:3156–66. doi: 10.1002/jsfa.13206, 38073022

[55] Xie Z Zeng Z Chen G Dong W Peng Y Xu W . Intracellular polysaccharides of aspergillus cristatus from fuzhuan brick tea leverage the gut microbiota and repair the intestinal barrier to ameliorate DSS–induced colitis in mice. J Agric Food Chem. (2023) 71:8023–37. doi: 10.1021/acs.jafc.3c00611, 37203140

[56] Yang X–Y Chen P–F He J–H. High consumption of sweetened beverages might increase the risk of inflammatory bowel diseases. Clin Gastroenterol Hepatol. (2019) 17:1417–8. doi: 10.1016/j.cgh.2018.11.053, 31126416

[57] Maniyar I Jajoo A Brewer GG Iyengar P Nguyen A Fasulo C . Dietary sugar and sweetened beverage intake increases inflammatory bowel disease risk: a systematic review and meta–analysis. United European Gastroenterol J. (2025) 13:1379–409. doi: 10.1002/ueg2.70047, 40532067 PMC12529010

[58] Fu T Chen H Chen X Sun Y Xie Y Deng M . Sugar–sweetened beverages, artificially sweetened beverages and natural juices and risk of inflammatory bowel disease: a cohort study of 121,490 participants. Aliment Pharmacol Ther. (2022) 56:1018–29. doi: 10.1111/apt.17149, 35848057 PMC9546432

[59] Ahsan M Koutroumpakis F Rivers CR Wilson AS Johnston E Hashash JG . High sugar–sweetened beverage consumption is associated with increased health care utilization in patients with inflammatory bowel disease: a multiyear, prospective analysis. J Acad Nutr Diet. (2022) 122:1488–1498.e1. doi: 10.1016/j.jand.2022.01.001, 34999242

[60] Shon W–J Jung MH Kim Y Kang GH Choi EY Shin D-M. Sugar–sweetened beverages exacerbate high–fat diet–induced inflammatory bowel disease by altering the gut microbiome. J Nutr Biochem. (2023) 113:109254. doi: 10.1016/j.jnutbio.2022.109254, 36572070

[61] Khalili H Hakansson N Chan SS Ludvigsson JF Olen O Chan AT . No association between consumption of sweetened beverages and risk of later–onset crohn’s disease or ulcerative colitis. Clin Gastroenterol Hepatol. (2019) 17:123–9. doi: 10.1016/j.cgh.2018.04.059, 29751165

[62] Piovani D Danese S Peyrin–Biroulet L Nikolopoulos GK Lytras T Bonovas S. Environmental risk factors for inflammatory bowel diseases: an umbrella review of meta–analyses. Gastroenterology. (2019) 157:647–659.e4. doi: 10.1053/j.gastro.2019.04.016, 31014995

[63] Gao Q Li J Feng J–R. Beverage consumption and risk of ulcerative colitis: a meta–analysis. Chinese J Gastroenterol Hepatol. (2018) 27:291–5. doi: 10.3969/j.issn.1006-5709.2018.03.011

[64] Han MK Anderson R Viennois E Merlin D. Examination of food consumption in United States adults and the prevalence of inflammatory bowel disease using national health interview survey 2015. PLoS One. (2020) 15:e0232157. doi: 10.1371/journal.pone.0232157, 32324818 PMC7179926

