# Supplementary material for: Association between different drinks consumption and risk of inflammatory bowel disease: a dose–response meta–analysis
Source: Front Nutr. 2026 May 20;13:1817418. doi: 10.3389/fnut.2026.1817418 (PMC13229699; doi:10.3389/fnut.2026.1817418)
Supplement: Supplementary file 1 [file Table_1.docx]

**SUPPLEMENTARY MATERIAL**

**Table of Contents**

**Supplementary Methods 1** – Database search strategy.

**Supplementary Figure S1** – Egger’s publication bias plot of Beverage.

**Supplementary** **Figure S2** – Egger’s publication bias plot of Alcohol.

# Supplementary Figure S3 – Egger’s publication bias plot of Coffee.

**Supplementary Figure S4** – Egger’s publication bias plot of Tea.

**Supplementary Figure S5** – Sensitivity analysis of Beverage.

**Supplementary Figure S6** – Sensitivity analysis of Alcohol.

**Supplementary Figure S7** – Sensitivity analysis of Coffee.

**Supplementary Figure S8** – Sensitivity analysis of Tea.

**Supplementary Table 1** – Rating details of NOS.

**Supplementary Table 2** – Rating details of AHRQ.

**Supplementary Table 3** – Dose-response relationship between Alcohol and IBD risk.

**Supplementary Table 4** – Dose-response relationship between Alcohol and IBD risk.

**Supplementary Table 5** – Dose-response relationship between Coffee and IBD risk.

**Supplementary Table 6** – Dose-response relationship between Tea and IBD risk.

**Supplementary Methods 1**

**Pubmed**

(((((((((((((((((((((((Inflammatory Bowel Diseases[MeSH Terms]) OR (inflammatory bowel disease[Title/Abstract])) OR (IBD[Title/Abstract])) OR (Colitis Ulcerative[MeSH Terms])) OR (ulcerative colitis[Title/Abstract])) OR (UC[Title/Abstract])) OR (Crohn Disease[MeSH Terms])) OR (Crohn's disease[Title/Abstract])) OR (Crohn disease[Title/Abstract])) OR (regional enteritis[Title/Abstract])) OR (Crohn's Enteritis[Title/Abstract])) OR (Regional Enteritis[Title/Abstract])) OR (Ileocolitis[Title/Abstract])) OR (Ileitis, Terminal[Title/Abstract])) OR (Terminal Ileitis[Title/Abstract])) OR (Ileitis, Regional[Title/Abstract])) OR (Regional Ileitides[Title/Abstract])) OR (Regional Ileitis[Title/Abstract])) OR (Enteritis, Granulomatous[Title/Abstract])) OR (Granulomatous Enteritis[Title/Abstract])) OR (Enteritis, Regional[Title/Abstract])) OR (Colitis, Granulomatous[Title/Abstract])) OR (Granulomatous Colitis[Title/Abstract])) AND (((((((((((((((((((beverage[Title/Abstract]) OR (tea[Title/Abstract])) OR (alcohol[Title/Abstract])) OR (wine[Title/Abstract])) OR (beer[Title/Abstract])) OR (liquor[Title/Abstract])) OR (coffee[Title/Abstract])) OR (soda[Title/Abstract])) OR (soft drinks[Title/Abstract])) OR (diet[Title/Abstract])) OR (carbonated beverage[Title/Abstract])) OR (soda pop[Title/Abstract])) OR (cola beverage[Title/Abstract])) OR (cola drink[Title/Abstract])) OR (cordial[Title/Abstract])) OR (cordial beverage[Title/Abstract])) OR (cordial drink[Title/Abstract])) OR (flavoured water[Title/Abstract])) OR (artificial juices[Title/Abstract]))

**Web of science**

(TS=("Inflammatory bowel disease" OR "IBD" OR "Ulcerative colitis" OR "Crohn's disease" OR "UC" OR "CD")) AND TS=(“Beverage” OR “tea” OR “alcohol” OR “wine” OR “beer” OR “liquor” OR “coffee” OR “soda” OR “soft drinks diet” OR “carbonated beverage” OR “soda pop” OR “cola beverage” OR “cola drink” OR “cordial” OR “Cordial” OR “beverage” OR “cordial drink” OR “flavoured water” OR “artificial juices”) AND Open Access

**Sinomed**

( ""炎症性肠病""[常用字段:智能] OR ""炎症性肠疾病""[常用字段:智能] OR ""溃疡性结肠炎""[常用字段:智能] OR ""克罗恩病""[常用字段:智能] OR ""克罗恩氏病""[常用字段:智能] OR ""局限性肠炎""[常用字段:智能] OR ""肉芽肿性肠炎""[常用字段:智能] OR ""肉芽肿性结肠炎""[常用字段:智能] OR ""回肠炎""[常用字段:智能] OR ""末端回肠炎""[常用字段:智能]) AND( ""饮料""[常用字段:智能] OR ""茶""[常用字段:智能] OR ""酒精""[常用字段:智能] OR ""酒""[常用字段:智能] OR ""葡萄酒""[常用字段:智能] OR ""啤酒""[常用字段:智能] OR ""烈酒""[常用字段:智能] OR ""咖啡""[常用字段:智能] OR ""苏打""[常用字段:智能] OR ""软饮料""[常用字段:智能] OR ""汽水""[常用字段:智能] OR ""可乐""[常用字段:智能] OR ""碳酸饮料""[常用字段:智能] OR ""果汁饮料""[常用字段:智能] OR ""人工果汁""[常用字段:智能])

**Cochrane library**

#1 inflammatory bowel disease OR Inflammatory Bowel Disease OR Colitis, Ulcerative OR Ulcerative Colitis OR regional enteritis OR bowel disease OR Inflammatory Bowel Disease OR Colitis, Ulcerative OR Ulcerative Colitis OR regional enteritis

#2 Beverage OR tea OR alcohol OR wine OR beer OR liquor OR coffee OR soda OR soft drinks diet OR carbonated beverage OR soda pop OR cola beverage OR cola drink OR cordial OR cordial OR beverage OR cordial drink OR flavoured water OR artificial juices

#3 #1 AND #2

**Embase**

(((((((((((((((((inflammatory AND ('bowel'/exp OR bowel) AND ('disease'/exp OR disease) OR inflammatory) AND ('bowel'/exp OR bowel) AND ('disease'/exp OR disease) OR 'colitis,'/exp OR colitis,) AND ulcerative OR ulcerative) AND ('colitis'/exp OR colitis) OR regional) AND ('enteritis'/exp OR enteritis) OR 'bowel'/exp OR bowel) AND ('disease'/exp OR disease) OR inflammatory) AND ('bowel'/exp OR bowel) AND ('disease'/exp OR disease) OR 'colitis,'/exp OR colitis,) AND ulcerative OR ulcerative) AND ('colitis'/exp OR colitis) OR regional) AND ('enteritis'/exp OR enteritis) AND ('beverage'/exp OR beverage) OR 'tea'/exp OR tea OR 'alcohol'/exp OR alcohol OR 'wine'/exp OR wine OR 'beer'/exp OR beer OR 'liquor'/exp OR liquor OR 'coffee'/exp OR coffee OR 'soda'/exp OR soda OR soft) AND drinks AND ('diet'/exp OR diet) OR carbonated) AND ('beverage'/exp OR beverage) OR 'soda'/exp OR soda) AND pop OR 'cola'/exp OR cola) AND ('beverage'/exp OR beverage) OR 'cola'/exp OR cola) AND drink OR 'beverage'/exp OR beverage OR cordial) AND drink OR flavoured) AND ('water'/exp OR water) OR artificial) AND juices

**Wiley**

"inflammatory bowel disease OR Inflammatory Bowel Disease OR Colitis, Ulcerative OR Ulcerative Colitis OR regional enteritis OR bowel disease OR Inflammatory Bowel Disease OR Colitis, Ulcerative OR Ulcerative Colitis OR regional enteritis" anywhere and "Beverage OR tea OR alcohol OR wine OR beer OR liquor OR coffee OR soda OR soft drinks diet OR carbonated beverage OR soda pop OR cola beverage OR cola drink OR cordial OR cordial OR beverage OR cordial drink OR flavoured water OR artificial juices" anywhere

**CNKI**

（主题：炎症性肠病 + 溃疡性结肠炎 + 克罗恩病）AND（主题：饮料 + 酒 + 茶 + 咖啡）

**万方学术期刊全文数据库**

(主题:(炎症性肠病 OR 溃疡性结肠炎 OR 克罗恩病) and 主题:(饮料 OR 酒 OR 茶 OR 咖啡))

**Supplementary Figures：**

| S1:Egger’s publication bias plot of Beverage.  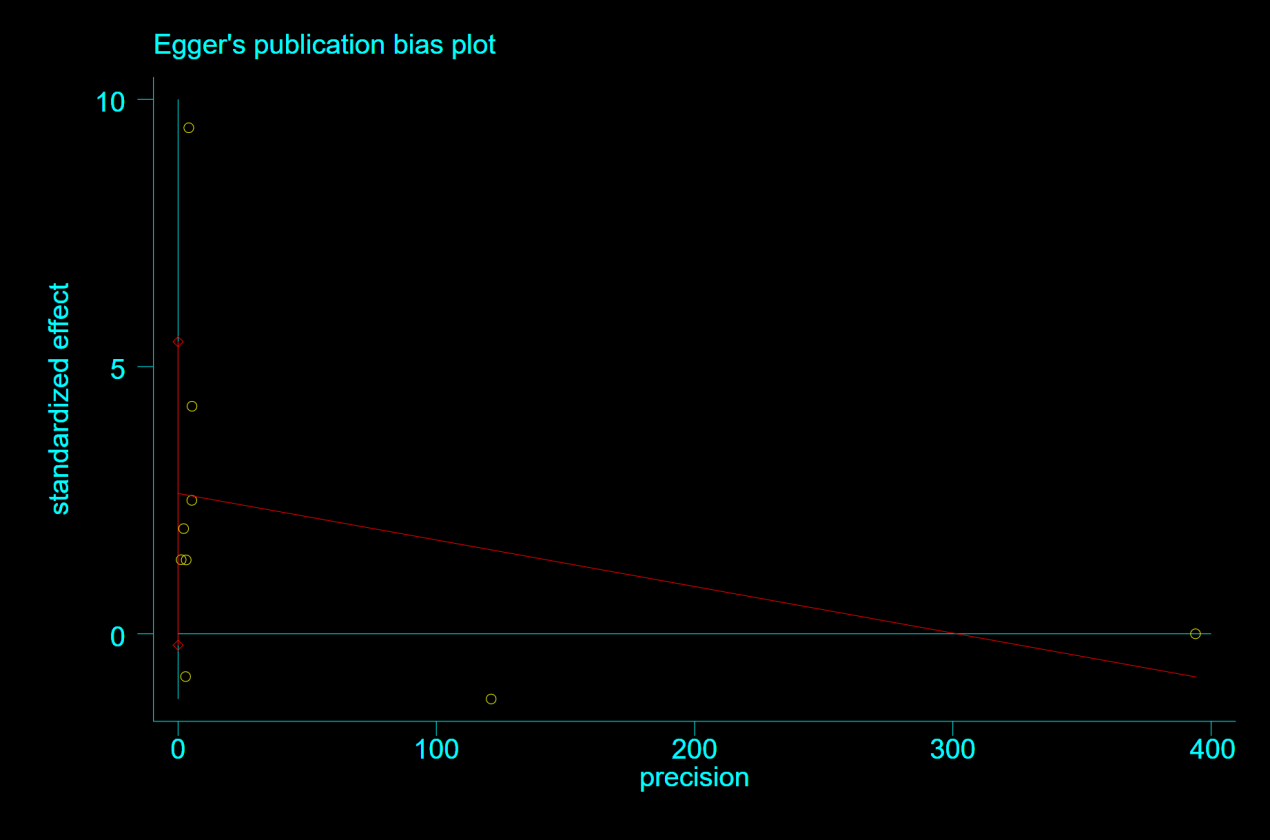 |
| --- |

| S2:Egger’s publication bias plot of Alcohol.  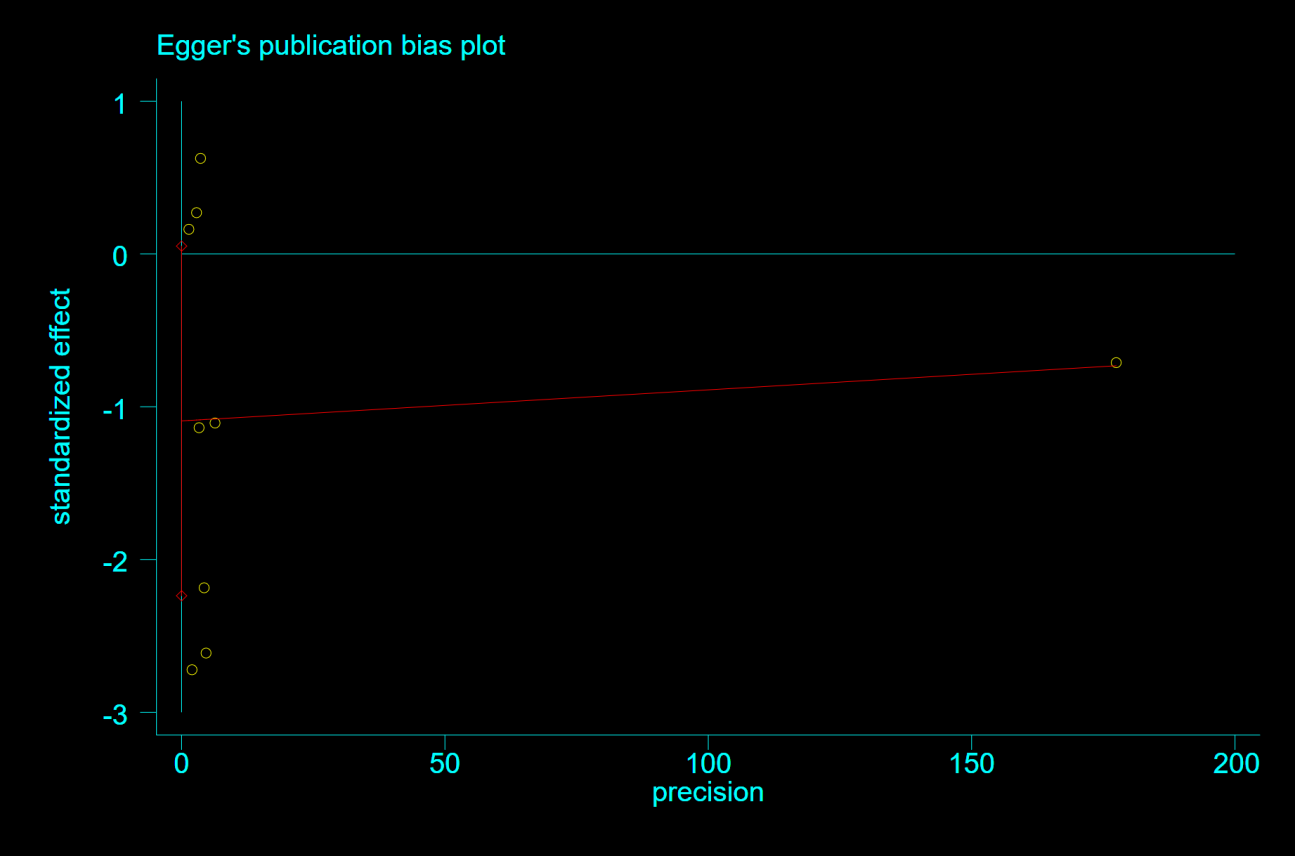 |
| --- |

| S3:Egger’s publication bias plot of Coffee.  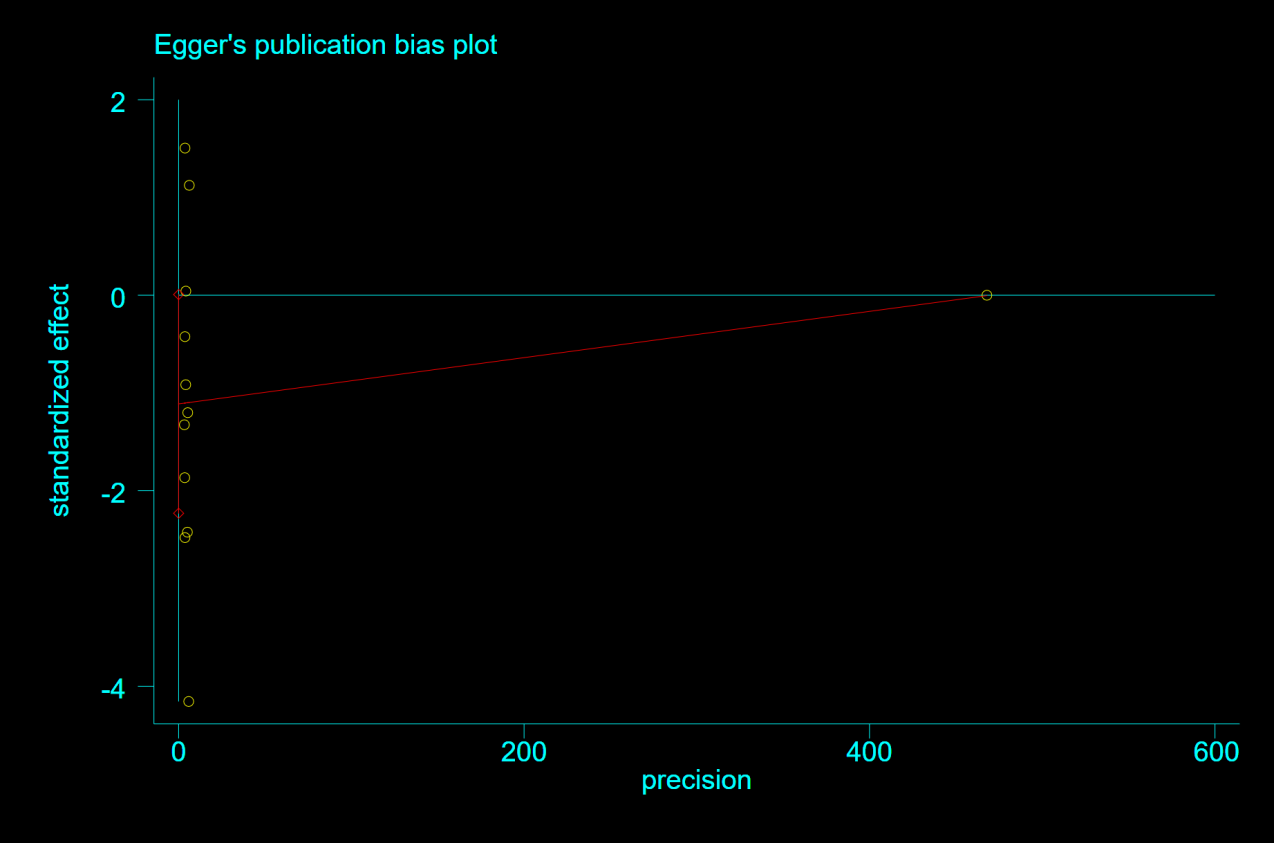 |
| --- |

| S4:Egger’s publication bias plot of Tea.  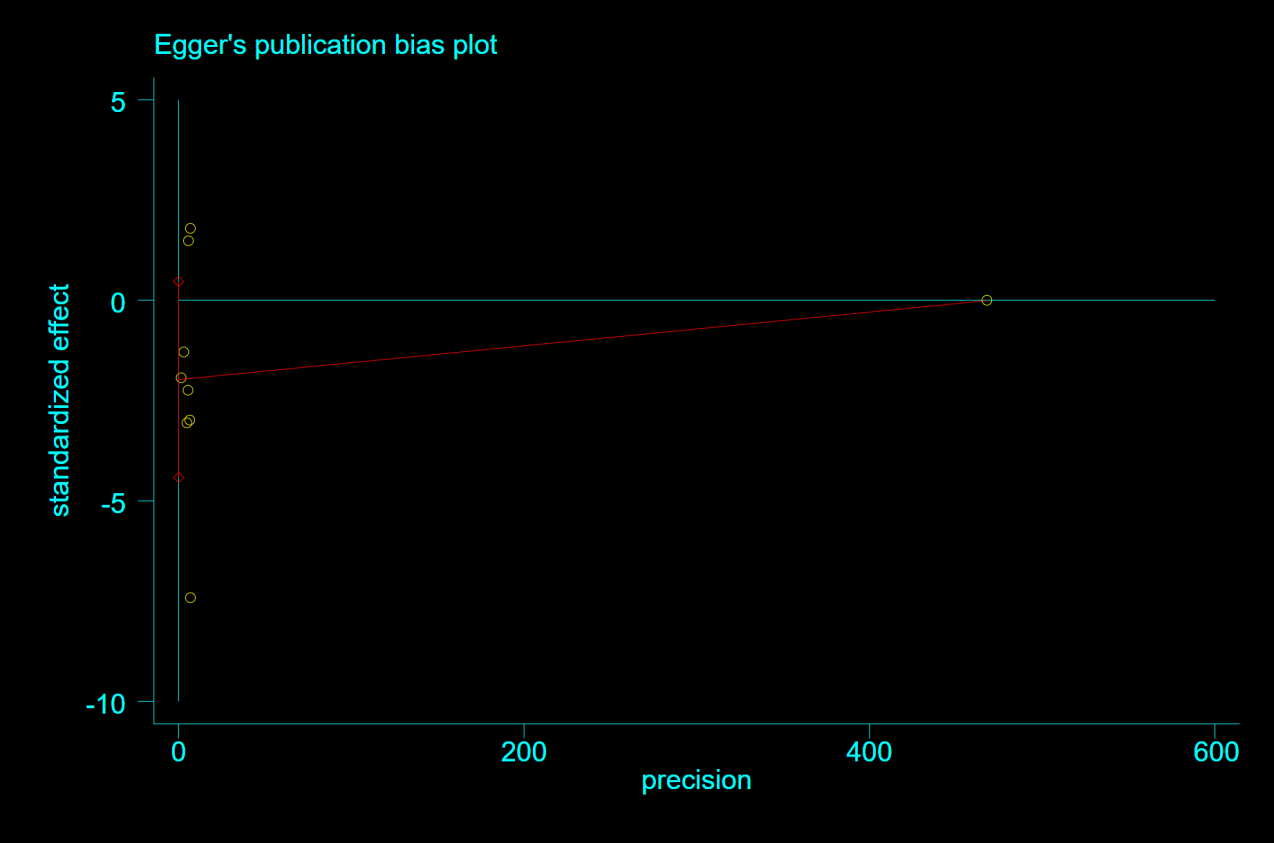 |
| --- |

| S5:Sensitivity analysis of Beverage.  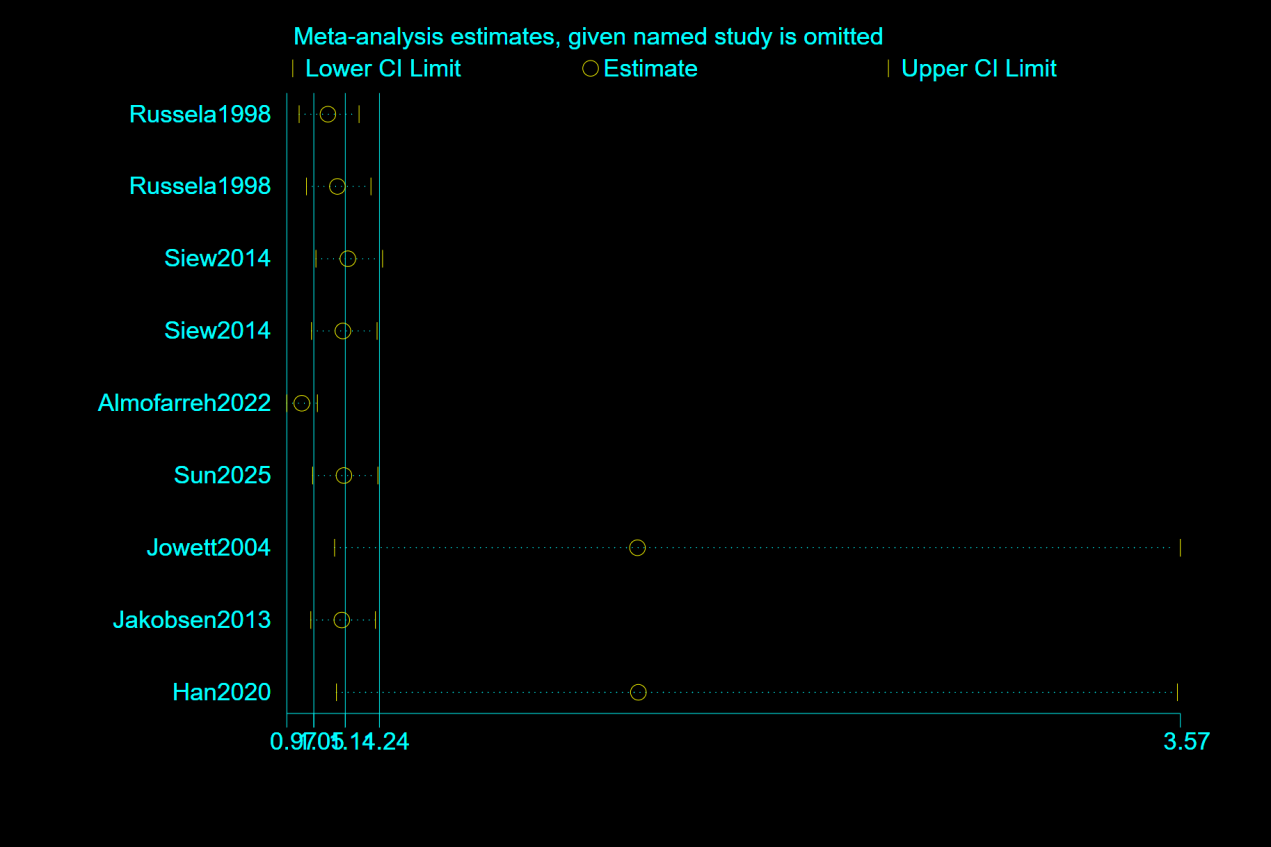 |
| --- |

| S6:Sensitivity analysis of Alcohol.  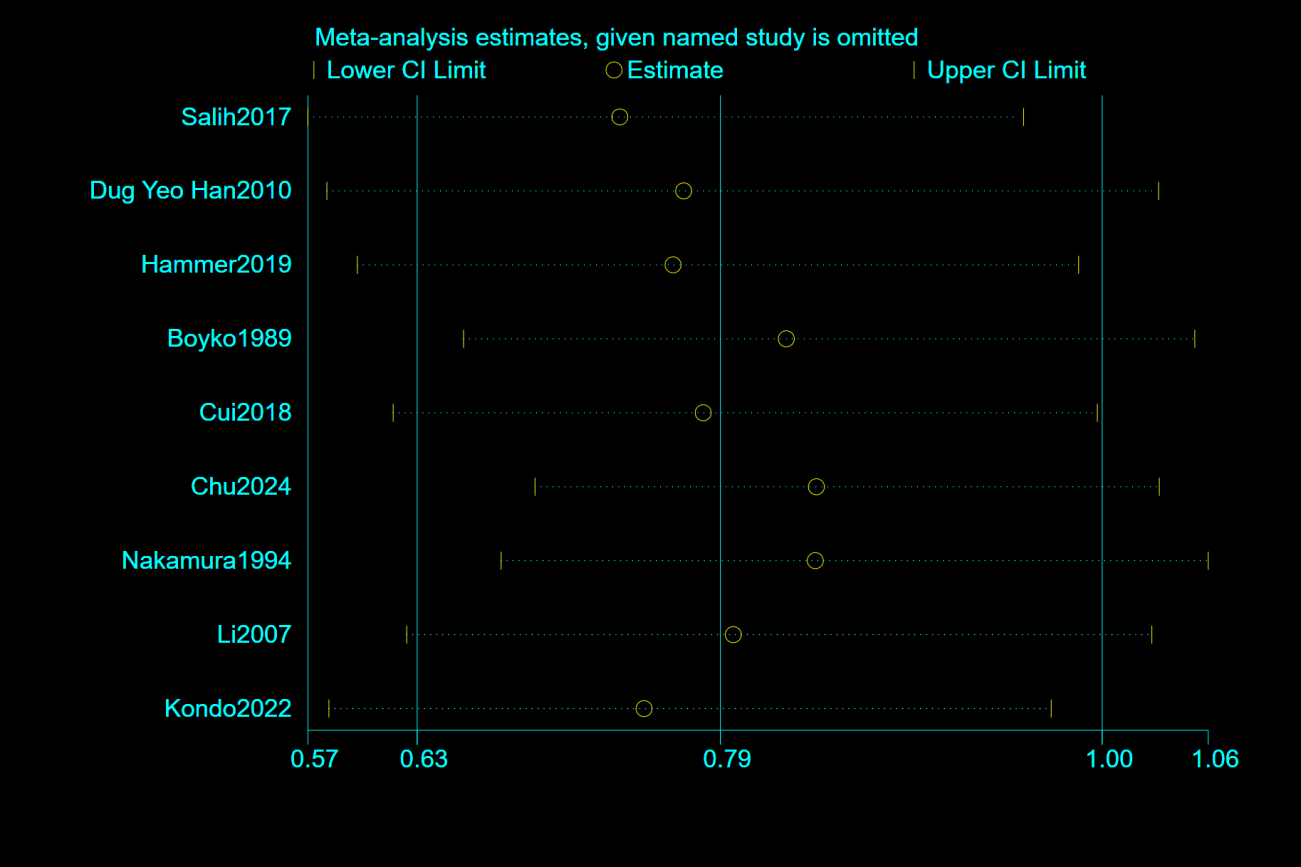 |
| --- |

| S7:Sensitivity analysis of Coffee.  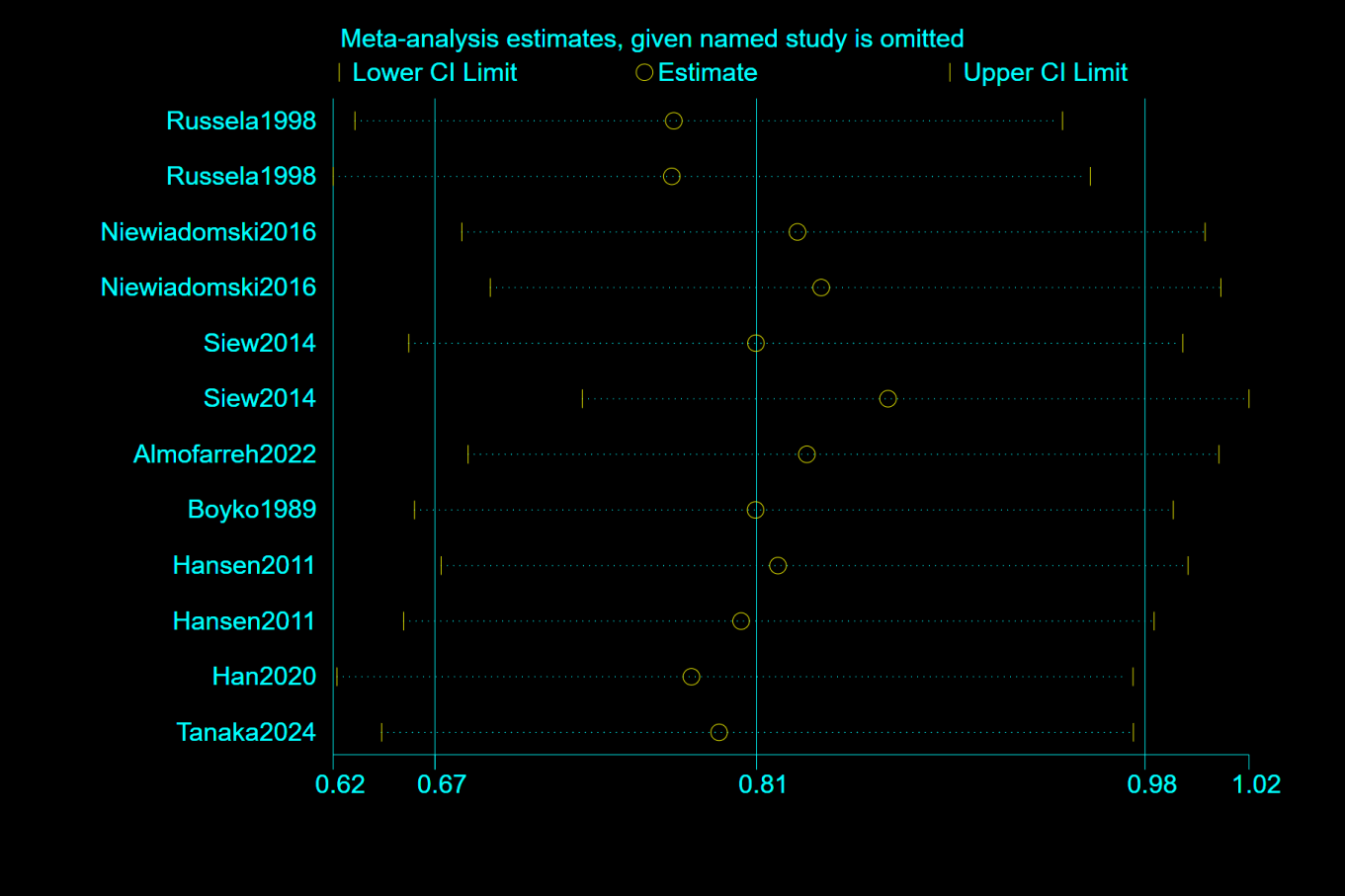 |
| --- |

| S8:Sensitivity analysis of Tea.  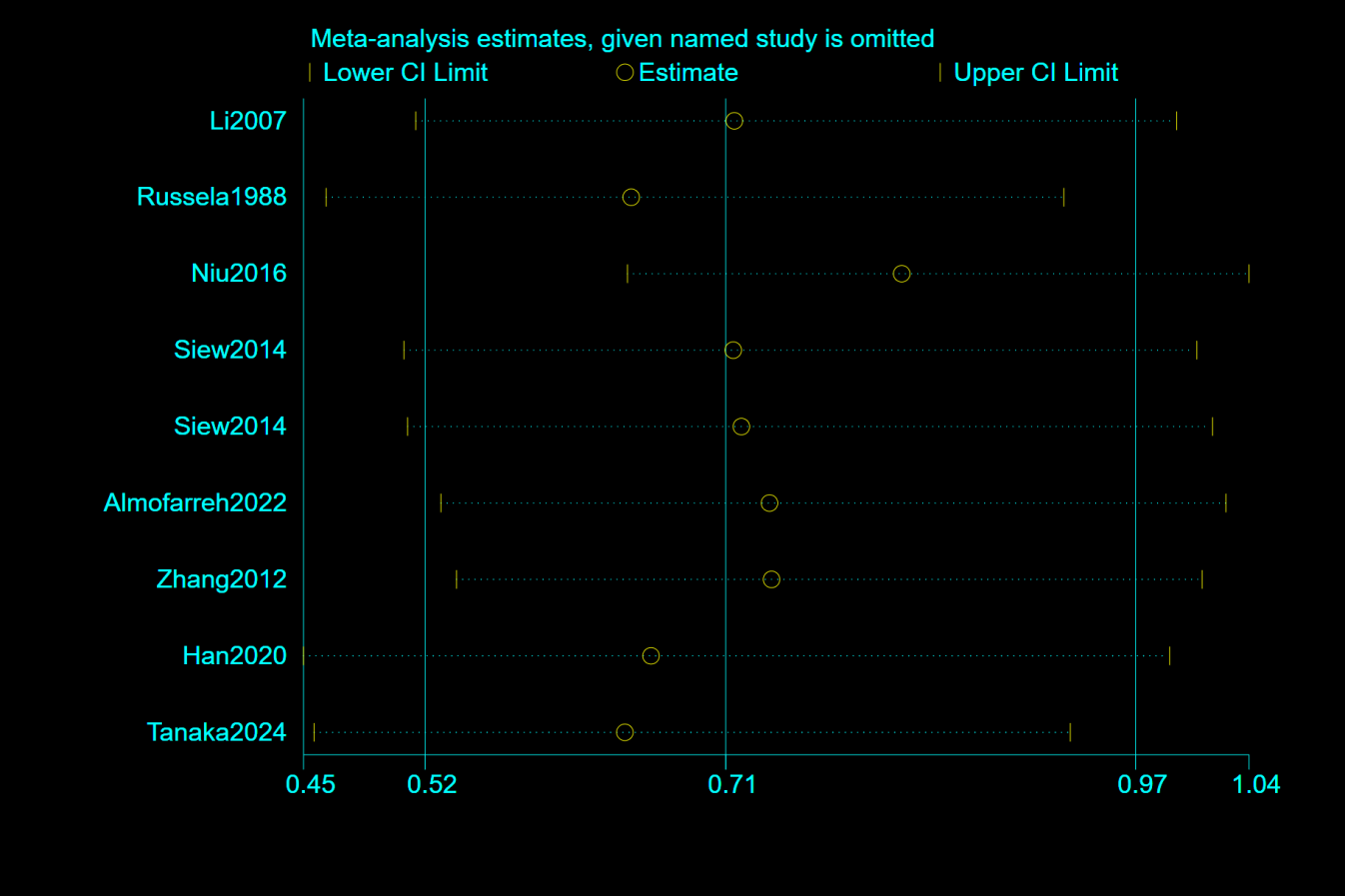 |
| --- |

Table 1 Rating details of NOS.

| Study Name | Selection | Comparability | Exposure | Quality Score |
| --- | --- | --- | --- | --- |
| Almofarreh2022 | 4/4 | 2/2 | 2/3 | 8 |
| Chu2024 | 4/4 | 2/2 | 2/3 | 8 |
| Boyko1989 | 3/4 | 2/2 | 2/3 | 7 |
| Cui2018 | 3/4 | 1/2 | 2/3 | 6 |
| DugYeoHan2010 | 4/4 | 2/2 | 2/3 | 8 |
| Zhang2012 | 3/4 | 1/2 | 2/3 | 6 |
| Hammer2019 | 4/4 | 1/2 | 3/3 | 8 |
| Jowett2004 | 4/4 | 2/2 | 2/3 | 8 |
| Jakobsen2013 | 4/4 | 2/2 | 2/3 | 8 |
| Hansen2011 | 4/4 | 2/2 | 2/3 | 8 |
| Nakamura1994 | 3/4 | 2/2 | 2/3 | 7 |
| Niewiadoski2016 | 3/4 | 2/2 | 2/3 | 7 |
| Li2007 | 4/4 | 1/2 | 2/3 | 8 |
| Kondo2022 | 4/4 | 2/2 | 2/3 | 8 |
| Russel1998 | 4/4 | 2/2 | 3/3 | 9 |
| Siew2014 | 4/4 | 2/2 | 2/3 | 8 |
| Niu2016 | 4/4 | 2/2 | 2/3 | 8 |
| Salih2017 | 4/4 | 2/2 | 2/3 | 8 |
| Bergmann2017 | 4/4 | 2/2 | 2/3 | 8 |
| Tanaka2024 | 4/4 | 2/2 | 3/3 | 9 |
| Sun2025 | 3/4 | 2/2 | 2/3 | 7 |
| Liu2022 | 4/4 | 2/2 | 2/3 | 8 |
| Hart2008 | 4/4 | 2/2 | 2/3 | 8 |
| Wang2013 | 4/4 | 2/2 | 2/3 | 8 |
| Sakamoto2005 | 4/4 | 1/2 | 2/3 | 7 |
| Persson1992 | 4/4 | 2/2 | 2/3 | 8 |

Table 2

Rating details of AHRQ.

| Study Name | 1 | 2 | 3 | 4 | 5 | 6 | 7 | 8 | 9 | 10 | 11 | Quality Score |
| --- | --- | --- | --- | --- | --- | --- | --- | --- | --- | --- | --- | --- |
| Han2020 | √ | √ | √ | × | × | √ | √ | √ | √ | √ | × | 8 |

Table 5

Dose-response relationship between Alcohol and IBD risk.

| Dose（grams/day） | exp(xb) | 95% CI |
| --- | --- | --- |
| 0 | 1.00 | 1.00-1.00 |
| 0.64999998 | 1.00 | 1.00-1.00 |
| 1 | 1.00 | 0.99-1.00 |
| 1.85 | 0.99 | 0.99-1.00 |
| 4.5 | 0.99 | 0.98-1.00 |
| 12.5 | 0.96 | 0.93-0.99 |
| 36 | 0.89 | 0.82-0.97 |
| 40 | 0.88 | 0.80-0.97 |
| 72 | 0.80 | 0.67-0.95 |
| 81.199997 | 0.77 | 0.64-0.94 |
| 192 | 0.55 | 0.34-0.87 |

Table 6

Dose-response relationship between Alcohol and IBD risk.

| Dose（cups/day） | exp(xb) | 95% CI |
| --- | --- | --- |
| 0 | 1.00 | 1.00-1.00 |
| 0.5 | 1.00 | 0.98-1.02 |
| 1 | 1.00 | 0.96-1.04 |
| 1.5 | 0.99 | 0.95-1.04 |
| 3.5 | 0.94 | 0.89-1.00 |
| 6 | 0.94 | 0.88-1.00 |

Table 7

Dose-response relationship between Coffee and IBD risk.

| Dose（grams/day） | exp(xb) | 95% CI |
| --- | --- | --- |
| 0 | 1.00 | 1.00-1.00 |
| 0.28600001 | 0.91 | 0.89-0.93 |
| 0.5 | 0.84 | 0.81-0.88 |
| 0.82499999 | 0.76 | 0.70-0.81 |
| 1 | 0.71 | 0.65-0.78 |
| 1.5 | 0.60 | 0.53-0.68 |
| 2.4000001 | 0.44 | 0.36-0.54 |
| 3.5999999 | 0.30 | 0.22-0.40 |

Table 8

Dose-response relationship between tea and IBD risk.

| Dose（times/week） | exp(xb) | 95% CI |
| --- | --- | --- |
| 0 | 1.00 | 1.00-1.00 |
| 0.5 | 1.15 | 1.06-1.23 |
| 1.2 | 1.18 | 1.07-1.30 |
| 1.5 | 1.14 | 1.04-1.26 |
| 2 | 1.06 | 0.95-1.18 |
| 3.5999999 | 0.89 | 0.75-1.06 |
| 4 | 0.87 | 0.72-1.04 |
| 6 | 0.82 | 0.67-0.99 |
| 8.3999996 | 0.84 | 0.61-1.15 |
